# Phylogenetic Relatedness Does not Predict Patterns of Parallel Transcriptome Adaptation in *Drosophila*

**DOI:** 10.1093/gbe/evaf161

**Published:** 2025-08-19

**Authors:** Julie M Cridland, Giovanni Hanna, Tiezheng Fan, David J Begun

**Affiliations:** Department of Evolution and Ecology, University of California, Davis, CA 95616, USA; Department of Evolution and Ecology, University of California, Davis, CA 95616, USA; Department of Evolution and Ecology, University of California, Davis, CA 95616, USA; Department of Evolution and Ecology, University of California, Davis, CA 95616, USA

**Keywords:** *Drosophila*, accessory glands, testis, gene expression, parallelism

## Abstract

Identifying the factors determining the repeatability of adaptation is a long-standing problem in evolutionary biology. Addressing this problem requires both comparative analysis and an understanding of how genetic variation within species responds to natural selection. Latitudinal clines are a classic system for studying adaptation in many species, including *Drosophila*. Here we investigate male reproductive tract (testis and accessory gland [AG]) transcriptomes from Maine (USA) and Panama City (Panama) populations of three species that have recently colonized North America, a pair of close relatives, *Drosophila melanogaster*, *Drosophila simulans*, and a much more distantly related species, *Drosophila hydei*. We observed strong evidence of parallel gene expression adaptation in the AG, but little such evidence for the testis. This parallelism takes the form of genes that exhibit high vs. low latitude expression differentiation in multiple species, as well as between-species correlations of high vs. low latitude log fold changes. However, the degree of parallelism among these species is not related to their relatedness. More specifically, *D. simulans*, which is very closely related to *D. melanogaster* yet very distantly related to *D. hydei*, shows much stronger parallelism for latitudinal AG transcriptome differentiation with the latter than with the former. This, despite the reproductive biology of *D. melanogaster* and *D. simulans*, is very similar and highly diverged from that of *D. hydei*. These results suggest that despite a signal of adaptive parallelism among all three species, the underlying selection responses are not well predicted by relatedness or similar ecologies, suggestive of idiosyncratic processes operating simultaneously with deterministic ones.

Significance Patterns of parallel adaptation have been studied in Drosophila on short evolutionary timescales by comparing the closely related species *Drosophila melanogaster* and *Drosophila simulans*. This study utilizes *Drosophila hydei*, a distantly related species with a similar history of recent expansion in North America. We find that incorporating this species results in a strong signal of adaptive parallelism in Drosophila and a surprising correlation in adaptation between more distantly related species.

## Introduction

Consistent geographical patterns of animal and plant phenotypic variation, such as latitudinal or altitudinal clines, are common (eg Haldane 1948; Endler 1977). Nevertheless, because such patterns can be the result of natural selection, demographic phenomena, or environmental effects, and because the connections between phenotypic variation, genetic variation, and fitness variation can be challenging to understand, the relative importance of selection vs. other phenomena, and the breadth of selective responses to spatially heterogeneous selection pressures across traits and species are open questions. In this context, the comparative analysis of clines across multiple species has two virtues. First, when a phenotypic cline with a genetic basis is observed in multiple species that do not share long-term correlated demographic histories, selection is the likely explanation because the probability of similarly structured demographic clines in organisms with no shared genetic variation or recent shared evolutionary histories is nil. A good example of such clines is repeated latitudinal variation for body size in vertebrates, a pattern so common that it is known as Bergmann's Rule (Bergmann 1847; Mayr 1956). Second, and related, the comparative analysis of clines speaks to the question of whether there are general rules of adaptation to variable environments and how such rules might vary over phylogeny or ecology. For example, in principle, two closely related species could exhibit latitudinal clines driven by selection, but the repeatability of the selection response might vary over phenotypes. Alternatively, species may exhibit shared, selectively driven phenotypic clines for the same traits, but the underlying genetics might be shared or not. The degree to which clines are shared across similarly selected species might depend on the complexity of standing variation on which selection acts, biological differences among species influencing the genetic and phenotypic details of the selection response, and stochasticity of the selection response. The relative importance of these factors generating similarities and differences between species in latitudinal clines is a topic of substantial interest.

The genus *Drosophila* has been a central model system in the study of clinal variation, as multiple species exhibit latitudinal or altitudinal clines (Dobzhansky 1944; Mettler et al. 1977; Singh et al. 1982; Gilchrist et al. 2004; Levitan and Etges 2005; Arthur et al. 2008; Allen et al. 2017). In some cases, clines for the same phenotype, such as body size, are observed in multiple species (Imasheva et al. 1994; Calboli et al. 2003; Hoffmann and Weeks 2007; Adrion et al. 2015; Fabian et al. 2015). More generally, however, because *Drosophila* evolutionary biologists working on clines have tended historically to measure a relatively small set of morphological or life history phenotypes and often measured in different ways in different laboratories, our view of the phenotypes responding repeatedly to spatially varying selection in Drosophila is limited and biased.

Among *Drosophila* species, *Drosophila melanogaster* clines have received the most attention, with a focus on North America and Australia (Hoffmann and Weeks 2007; Adrion et al. 2015). This species originated in Africa, colonized Europe around 10,000 years ago and only recently—within the last 200 years—colonized North America and Australia (David and Capy 1982; Pool et al. 2012). Several morphological and life history traits exhibit clines on one or both continents (Hoffmann and Weeks 2007; Adrion et al. 2015). Similarly, the species exhibits extensive clinal genomic differentiation, often on both continents (Reinhardt et al. 2014; Schrider et al. 2016). Thus, this species provides a promising arena for studying rapid adaptation to spatially heterogeneous environments. Interestingly, the sister species, *Drosophila simulans*, which appears to have originated in Madagascar (Dean and Ballard 2004) and also only recently colonized the rest of the world (Capy and Gibert 2004), exhibits much less clinality than *D. melanogaster*, possibly because its colonization of the Americas and Australia may be more recent than that of *D. melanogaster* (Capy and Gibert 2004). Conclusions about the degree of clinality of *D. simulans*, however, are less secure given that it has received much less attention than *D. melanogaster*.

The possible role of geographic gene expression differentiation in local adaptation has recently been investigated on a transcriptomic scale in several animal and plant taxa (Levine et al. 2011; Fraser 2013; Morris et al. 2014; Dayan et al. 2015; Svetec et al. 2015; Juneja et al. 2016; Allen et al. 2017; Mack et al. 2018; Rivas et al. 2018; Ravindran et al. 2019; Huang et al. 2020; Jacobs et al. 2020; Blanc et al. 2021). Gene expression phenotypes are appealing in the context of local adaptation in multiple ways. First, thousands of phenotypes are measured simultaneously. Second, these expression phenotypes can generate interesting new hypotheses on downstream phenotypes or biological processes that might be targets of selection or be influenced by such selection. Third, at least in some contexts (eg cis-acting regulatory variation), expression phenotypes may be somewhat more proximate to the underlying genetics compared with classic quantitative traits, which creates opportunities to make connections between expression phenotypes and the regulatory mechanisms and population-level processes that might influence them.

In previous work (Zhao et al. 2015), we investigated latitudinal gene expression differentiation in recently established North American populations of *D. melanogaster* and *D. simulans* using whole male transcriptome data from populations sampled from Panama City (Panama) and Fairfield (Maine, USA). That study revealed extensive latitudinal expression differentiation for both species. While the number of genes exhibiting strong latitudinal expression differentiation was small, it was more than expected by chance, thereby supporting the proposition that a component of latitudinal expression differentiation was the result of parallel selection responses. We observed no enrichment of testis-biased or testis-specific genes among the differentially expressed (DE) genes. Similarly, whole male latitudinal transcriptome differentiation was observed in two other distantly related species, *Drosophila hydei* and *Drosophila serrata* (Allen et al. 2017; Zhao and Begun 2017), suggested the possibility that *Drosophila* genes showing geographic expression differentiation are not a random sample of whole male transcriptomes, but rather, are more similar than expected by chance. While this work was suggestive, whole animal transcriptome data almost certainly lead to underestimates of the number of DE genes at the tissue or organ level (Chintapalli et al. 2007) and provide little insight into possible differences in the relative importance of selection acting on expression phenotypes in different tissues and their underlying biology.

More recently, we continued our investigation of geographic differential expression in *D. melanogaster* males through the analysis of accessory gland (AG) + anterior ejaculatory duct (AG) and testis transcriptomes in Maine, Panama, and Zambia populations (Cridland et al. 2023). That work revealed that the Maine and Panama *D. melanogaster* males exhibit considerable geographic expression differentiation in the AG but little in the testis. That work also suggested that selection—primarily in Panama—contributed to the recent geographic differentiation of AG transcriptomes in these American *D. melanogaster* populations. We then extended the work of Zhao et al. (2015) on whole males by investigating parallel latitudinal transcriptome differentiation of AG and testis in *D. melanogaster* and *D. simulans* (Fan et al. 2025). While that study revealed no enrichment of shared latitudinally DE genes in the two species in either organ, we observed a highly significant correlation of latitudinal log fold change for the two species in the AG. We inferred from this observation that even small latitudinal differences in AG transcript abundance have been shaped by spatially varying selection in a similar way in the two species. Interestingly, there was no evidence of such a phenomenon in the testis.

Our goal here is to return to open questions regarding the extent of shared latitudinal expression differentiation in three focal species, *D. melanogaster*, *D. simulans*, and *D. hydei*, that have recently colonized North America, continuing our focus on two male reproductive tissues, the testis and AG. Specifically, here we extend our investigation of transcriptome local adaptation and interspecific parallelism to *D. hydei*. *Drosophila hydei*, a member of the repleta group, is likely South American ancestrally (Patterson and Stone 1952; Oliveira et al. 2012). Historical evidence supports the idea that, as is the case for *D*. *melanogaster* and *D. simulans*, colonization of high-latitude North American regions is very recent (Sturtevant 1921). While *D. melanogaster* and *D. simulans* shared a recent common ancestor (2 to 3 million years ago—Obbard et al. 2012), and share their basic reproductive biology, *D. hydei* diverged from the *D. melanogaster*/*D. simulans* lineage roughly 50 million years ago (Powell 1997; Tamura et al. 2004), and its reproductive biology is very different from that of the *melanogaster* subgroup. For example, *D. hydei* has evolved giant sperm and testis (Pitnick and Markow 1994), exhibits extraordinarily high female re-mating rates (Markow 1985), has lost the Sex Peptide gene, a key seminal fluid protein gene in *D. melanogaster* (McGeary and Findlay 2020; Hopkins and Perry 2022), and has many more secondary cells in the AG than *D. melanogaster* and *D. simulans* (Takashima et al. 2023).

In this report, we address three major questions in a three-species comparative population analysis. First, do the two male reproductive tract tissues, AG and testis, show similar patterns of differential expression across all three species—in other words, how strong is the tissue effect compared with the species effect on latitudinal expression differentiation? Second, how does evidence of parallel expression adaptation at the gene level manifest across the two tissues? Finally, given that *D. simulans* and *D. melanogaster* are much more closely related to each other than either is to *D. hydei*, do they exhibit more latitudinal parallelism with each other than either does with *D. hydei*?

## Results

### Differential Gene Expression in High vs. Low Latitude Populations

We measured gene expression—in median transcripts per million (TPM)—for replicated population pools of *D. melanogaster*, *D. simulans,* and *D. hydei* from Fairfield, Maine (USA) and Panama City, Panama, for each of two tissues, testis and AG (Table S1). As expected (Drosophila 12 Genomes Consortium et al. 2007; Graveley et al. 2010; Cridland et al. 2023), the testis expressed more genes—between 26% and 40% more—than the AG for all three species (Table S2). We ascertained the number of DE genes between Maine and Panama for each species (Table 1). Since our primary interest here is DE parallelism between species, we relaxed our typical *P*-value cutoff of 0.05 to a *P*-value cutoff of 0.1, with the logic being that a slightly less restrictive false discovery rate should not generate spurious parallelism.

**Table 1:** Differentially expressed genes

Species	Tissue	Total DE genes	Expressed genes	Percent DE
*D. melanogaster*	AG	798	8,023	9.9
*D. simulans*	AG	928	8,299	11.2
*D. hydei*	AG	1,568	7,687	20.4
*D. melanogaster*	Testis	39	10,888	0.4
*D. simulans*	Testis	757	10,483	7.2
*D. hydei*	Testis	472	10,433	4.5

We found consistently more DE genes (number and proportion) in the AG than in the testis. One possible explanation for this finding is weaker stabilizing selection on AG transcriptomes than on testis transcriptomes. However, the results presented below do not support this interpretation. We observed more DE genes in both *D. simulans* and *D. hydei* than in *D. melanogaster* for both tissues. While the differences among species in the proportion of DE genes are considerable for both tissues, it is especially stark for the testis, for which *D. melanogaster* and *D. simulans* exhibit the greatest difference, nearly 20-fold, despite the fact that they are the sister species pair. Among the species/tissue DE estimates, the *D. hydei* AG exhibits the greatest proportion (20%) of DE genes, much more than either *D. simulans* or *D. melanogaster*. Given that in all species the AG main cells constitute a large majority of the three presumptive cell types in the bulk dissection analyzed here (Takashima et al. 2023; Majane et al. 2024), elevated latitudinal DE in *D. hydei* main cells rather than latitudinal variation in the proportions of cell types in the bulk dissection (main cells, secondary cells, and ejaculatory duct cells) is the most likely explanation of the roughly 2-fold greater AG transcriptome DE in *D. hydei* compared with the other two species. The species rank order of proportion DE genes differs for the two tissues, revealing evidence of a tissue × species interaction. The rank order for testis is *D. simulans* > *D. hydei* > *D. melanogaster*; while for AG it is *D. hydei* > *D*. *simulans* > *D. melanogaster*. Focusing strictly on the 1:1:1 orthologs reveals the same rank orders (Table S3). By this criterion, *D. melanogaster* exhibits less expression differentiation than the other two species. These organ-based results are consistent with our previous result (Zhao et al. 2015), that *D*. *simulans* exhibits more whole male transcriptome differentiation between Maine and Panama than does *D. melanogaster*.

The discordance between phenotypic and genetic differentiation is notable. For example, while *D. simulan*s exhibits substantially greater expression differentiation than *D. melanogaster*, *D. simulans* shows substantially weaker genomic patterns of latitudinal differentiation than *D. melanogaster* (Machado et al. 2016; Sedghifar et al. 2016). Similarly, while *D. hydei* and *D. melanogaster* exhibit similar magnitudes of latitudinal genomic differentiation (as estimated by Maine vs. Panama Fst) (Zhao and Begun 2017), *D. hydei* exhibits much more latitudinal expression differentiation than *D. melanogaster* for both tissues. Discordant patterns of phenotypic and genomic differentiation are suggestive of a role for natural selection in driving phenotypic differentiation.

### Gene-Level Parallel Expression Differentiation

A major objective of this work was to investigate gene-level parallel latitudinal expression differentiation among three species that recently colonized high-latitude North American environments. To address this question, we limit our analysis to orthologs from pairwise species comparisons and the three-species orthologs (Tables 2, S4, and S5). While we did not observe an enrichment of shared DE genes amongst orthologs for *D. melanogaster* vs. *D. simulans* (Fan et al. 2025) or *D. melanogaster* vs. *D. hydei* for either tissue, we observed a highly significant excess of shared DE genes for both the AG and testis for *D. simulans* vs. *D. hydei*. In addition, we observed an excess of genes—14 observed vs. 7.6 expected—that show DE in the AG of all 3 species (Table S6). The AG exhibited a 1.7-fold enrichment (binomial *P*-value = 4.2e^−7^) of shared *D*. *simulans–D. hydei* DE orthologs, while the testis exhibited 1.6-fold enrichment (binomial *P*-value 3.48e^−3^), though with only 25 shared DE genes, this enrichment is not as convincing as that of the AG. Given that we previously found no evidence of testis contamination of the our AG dissections for either *D. simulans* or *D. melanogaster* (Cridland et al. 2023; Fan et al. 2025), we cannot identify an obvious artifact that could generate such shared gene enrichments, and thus, we conclude that spatially varying selection has influenced geographic transcriptome differentiation of these two species in a similar manner, with especially strong evidence in the AG. Surprisingly, the parallel latitudinal selection response appears to be much greater for two species that diverged roughly 30 to 50 million years ago than for two species that diverged only 2 to 3 million years ago (Obbard et al. 2012).

**Table 2:** Shared DE genes between species

Species 1	Species 2	Species 3	Tissue	Observed shared DE genes	Expected shared DE genes	Binomial test; *P*-values	% Higher expression in Maine	% Same directionality	% Same direction all genes	Binomial test; *P*-values
*D. melanogaster*	*D. simulans*	…	AG	60	64.2	6.74E−01	0.550	68	64.2	8.78E−02
*D. melanogaster*	*D. hydei*	…	AG	63	70.9	8.11E−01	0.556	68	61.1	5.35E−02
*D. simulans*	*D. hydei*	…	*AG*	214	151.1	4.18E−07	0.636	91	68.9	4.47E−15
*D. melanogaster*	*D. simulans*	*D. hydei*	AG	14	7.6	1.12E−02	0.571	71	46.8	4.04E−02
*D. melanogaster*	*D. simulans*	…	Testis	3	1.4	5.11E−02	0.333	33	46.9	3.97E−01
*D. melanogaster*	*D. hydei*	…	Testis	1	0.6	1.12E−01	0.000	0	47.3	5.27E−01
*D. simulans*	*D. hydei*	…	Testis	25	14.3	3.48E−03	0.400	64	49.5	5.67E−02
*D. melanogaster*	*D. simulans*	*D. hydei*	Testis	0	0.0	NA	NA	NA	NA	NA

In principle shared DE genes could show opposite directionality, which could blunt the argument that sharing results from correlated selection responses. To address this possibility, we investigated shared directionality among the shared DE genes. In the AG, we observed substantial agreement between species comparisons for shared directionality (Tables 2 and S7); relative to all AG-expressed genes, all three species exhibit a trend for shared DE genes to have a greater proportion of genes with higher expression in Maine than Panama, which represents another form of parallelism. This pattern is especially strong in the species pair showing the greatest amount of DE parallelism, as 91% of shared *D. simulans–D. hydei* DE orthologs also share directionality (significantly more than the proportion of all genes; binomial *P*-value = 4.47e^−15^), strongly supporting the idea that the *D. simulans/D. hydei* parallelism is selectively driven. Because the *D. melanogaste*r testis exhibits so few DE genes, we are only able to assess the shared testis DE genes between *D. simulans* and *D. hydei.* Here we see 64% of shared DE genes exhibit shared directionality, consistent with the pattern observed in the AG, though not significantly more than expected compared with all orthologs (binomial *P*-value = 0.057). Interestingly, for all three species, AG-expressed genes exhibited a deviation from the null expectation (ie no latitudinal parallelism)—that 50% of genes would have the same sign logFC between species pairs (Table S7). Instead, we observed that between 61% and 69% of AG-expressed genes have the same sign logFC between species pairs. In contrast, testis transcriptome expression directionality was not significantly different from the null expectation.

**Fig. 1.**
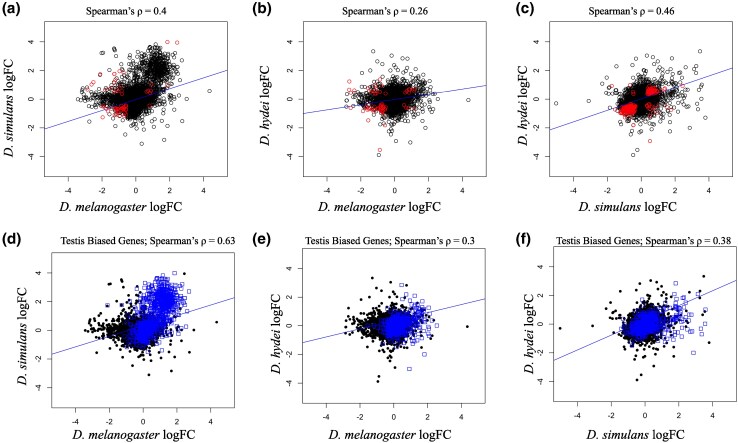
Pairwise comparisons of logFC between species in the AGs. For a–c) shared DE genes are in red, all other genes are in black. *P*-values for all comparisons are in Table S10, all values are <<0.05. a) *Drosophila melanogaster* vs. *D. simulan*s, b) *D. melanogaster* vs. *D. hydei*, c) *D. simulan*s vs. *D. hydei*. For d–f) testis-biased genes are in blue, all other genes are in black. d) *Drosophila melanogaster* vs. *D. simulan*s, e) *D. melanogaster* vs. *D. hydei*, f) *D. simulan*s vs. *D. hydei*.

To investigate more comprehensively the apparent pattern of parallel expression differentiation for whole transcriptomes, following Fan et al. (2025), we calculated Spearman's *ρ* to compare log fold changes for high vs. low latitude populations for all expressed orthologs in each tissue/species comparison. This analysis revealed a significant correlation between all three species pairs for the AG (Table S8, Fig. 1a–c; *P*-values << 0.01), consistent with the asymmetric expression directionality observed for all three species noted above. For all pairwise comparisons in the testis, *ρ* values were very close to 0 (Fig. S1a–c). The strength of the AG logFC correlations varied substantially, however: *D. melanogaster* vs. *D. hydei* had the weakest correlation (*ρ* = 0.26). The comparisons involving *D. simulans* were both substantially stronger with *ρ* = 0.4 compared with *D. melanogaster* and *ρ* = 0.46 compared with *D. hydei*. These observations further support the conclusion that *D. simulans* and *D. hydei*, two distantly related species with quite different reproductive biology and mating systems, exhibit strong parallel adaptation not just for the genes that are formally DE, but also for many genes that are expressed at different levels but are not individually significant. Thus, parallel local adaptation for the AG transcriptome in these two species appears to be broadly distributed across many genes. Overall, the strength of the expression correlations is unrelated to phylogenetic relatedness.

We sought evidence for connections between shared *D. simulans–D. hydei* DE genes in the AG and the AG single-cell marker genes identified in *D. melanogaster* (Majane et al. 2024). Forty-six single-cell markers (of 346 single-cell markers having *D. simulans–D. hydei* 1:1 orthologs) were significantly DE in the bulk tissue analyses presented here; 9 (9/76; 12%) main cell markers, 12 (12/56; 21%) secondary cell markers, and 25 (25/214; 12%) ejaculatory duct cell markers. This is intriguing because the main cells are the majority of cells in the bulk dissection (Takashima et al. 2023; Majane et al. 2024), yet they are the minority of differentiated markers. This apparent under-representation of DE main cell markers in these two species is consistent with the small number of *Sfps* that are DE in both *D. simulans* and *D. hydei* (given that many *D. melanogaster* main cell markers are *Sfps*). Moreover, these observations suggest that a significant component of the AG parallel adaptation in these two species occurs in secondary and ejaculatory duct cells, though single-cell analysis of the *D. hydei* AG will be required to put this conclusion on firmer footing.

### Parallel Transcription Factor Expression Differences in the AG

While the genetics of AG expression appears to be complex in *D. melanogaster* (Cridland et al. 2023) and is likely similarly complex in most species (Huang et al. 2015; GTEx Consortium 2020), we sought evidence for possible parallelism for trans-acting factors in all three species that could potentially contribute to expression parallelism. Using the *D. melanogaster* annotation, we identified 2,759 genes associated with the term “transcription factor.” Between *D. melanogaster* and *D. simulans,* we find five shared DE transcription factors (Table S9), including *tim*, which was previously identified in *D. melanogaster* (Cridland et al. 2023) at a more stringent *P* < 0.05 cutoff. All five, *tim*, *Abd-B*, *Taf3*, *CrebA*, and *Pdp1*, were more lowly expressed in Panama for both species. Between *D. melanogaster* and *D. hydei* only one transcription factor, *tim*, exhibited shared DE. Intriguingly, both *timeless* and *Pdp1* are circadian rhythm genes, suggesting that some latitudinal expression variation is connected to peripheral clocks relating to day length and/or seasonality differences between Maine and Panama. Interestingly, *Abd-B* and *CrebA* regulate key functions of the AG; *Abd-B* regulates secondary cells (Gligorov et al. 2013), while *CrebA* regulates secretion machinery (Johnson et al. 2020).

Ten transcription factors exhibited DE in the AG of both *D. simulans* and *D. hydei*: *cbd*, *crol*, *tou*, *Jra*, *tim*, *Hipk*, *Glut4EF*, *MEP-1*, *Mad*, and *lilli*. Eight of these were DE in the same direction, with lower expression in Panama in both species. There is little information connecting these transcription factors with AG function, though *Mad* regulates the bone morphogenetic protein (BMP) signaling pathway, which functions in *D. melanogaster* secondary cell biology (Leiblich et al. 2012; Corrigan et al. 2014; Redhai et al. 2016). As noted above, *tim* also exhibits DE in the *D. melanogaster* AG (Cridland et al. 2023) and is the only transcription factor showing DE in all three species AG; all three species show lower expression of *tim* in Panama.

### Tissue Bias and Differential Expression

As described in Materials and Methods, we used FlyAtlas2 (Leader et al. 2018; Cridland et al. 2023) data from *D. melanogaster* to calibrate an organ vs. whole male expression metric that we could apply uniformly to all three species (Table S10). Using this approach we observed many more testis-biased than AG-biased genes in all three species (Table S11), consistent with the literature (Graveley et al. 2010). All three species showed significant enrichment of DE of AG-biased genes in the AG, most extremely in *D. melanogaster,* where the enrichment was more than 2-fold. While this pattern could be explained by either spatially varying selection on, or weaker stabilizing selection on AG-biased genes than on other genes, the interspecific gene-level latitudinal expression parallelism noted above supports the former explanation. In contrast, the testis-biased genes were not significantly enriched for testis DE in either *D*. *melanogaster* or *D. hydei*, and showed only a slight enrichment (1.25-fold) of DE genes in *D. simulans* (Fisher's exact test, *P*-value = 0.005). Thus, despite some differences between species, the broad patterns of tissue bias and its correlation with latitudinal DE tend to be shared. Note, however, that the potentially different relative sizes of the AG and testis vs. whole male for *D. hydei* compared with *D. melanogaster* and *D. simulans* means that comparisons of tissue-bias patterns between *D. hydei* and the other species should be treated cautiously.

### Testis-Biased Genes Expressed in AGs

Fan et al. (2025) reported that the AG transcriptomes of *D. melanogaster* and *D. simulans* showed a stronger latitudinal logFC correlation for testis-biased genes than for non-testis-biased genes (categorizing tissue bias using all *D. melanogaster* male tissues in FlyAtlas2). We investigated this phenomenon further in the context of three pairwise species comparisons, using our organ vs. whole male estimates (as detailed tissue-level data are only available for *D*. *melanogaster*). For each species comparison, genes were categorized as tissue-biased if they were tissue-biased in either one or both species (Table S12). We recovered the same result as (Fan et al. 2025) for the *D. melanogaster–D. simulans* logFC, a strong AG transcriptome correlation for testis-biased genes and a weak correlation for the rest of the transcriptome. *Drosophila melanogaster–D. hydei* logFC correlations in the AG were similar across gene bias categories. Testis-biased orthologs showed a modestly stronger correlation than other non-testis-biased orthologs (Spearman's *ρ*, 0.3 vs. 0.25). In contrast, the *D. simulans–D. hydei* logFC correlations showed the opposite pattern with a somewhat higher correlation for non-testis-biased orthologs (Spearman's *ρ* 0.38 vs. 0.46). Overall correlations were similar across gene categories.

A subset of 91 orthologs were identified as AG-expressed, testis-based genes in all 3 species (Table S13). Within this set, logFC was highly correlated between *D. melanogaster* and *D. simulans* (*ρ* = 0.67, *P*-value ∼ 0), but not correlated for *D. melanogaster* vs. *D. hydei* (*ρ* = 0.18, *P*-value = 0.08) and modestly correlated for *D. simulans* vs. *D. hydei* comparisons (*ρ* = 0.21, *P*-value = 0.0497).

Overall, these results suggest that parallel DE for testis-biased genes in the AG is primarily a *D. melanogaster–D. simulans* phenomenon. Our results also suggest that of the three species, *D. simulans* exhibits the strongest selection response to latitudinal variation, as it shares components of adaptive parallelism with both *D. melanogaster* and *D. hydei*; moreover, the components of the AG parallelism with each of the two species are not highly similar to each other.

### Lineage-specific Expression Divergence in *D. simulans*

We previously used branch length variation to investigate lineage (population)-specific expression divergence (LED) in *D. melanogaster* (Cridland et al. 2023), which revealed that latitudinal DE in the AG was strongly associated with longer branch lengths to Panama than to Maine, suggestive of a selectively driven reduction in transcript abundance in Panama. We repeated this analysis here in *D. simulans* using Africa and Madagascar population samples to root our network of North American samples (Tables 3 and S1). The *D. simulans* testis transcriptome exhibits equally long branches to Maine and Panama. However, the AG exhibits significantly longer branch lengths to Panama than to Maine (0.51 vs. 0.39, *t*-test *P*-value 6.75e^−45^), similar to previous results from *D*. *melanogaster* (Cridland et al. 2023), and representing another form of parallel phenotypic evolution. Greater branch length heterogeneity in the AG is consistent with our finding of a skew in directionality in the AG of all three species, indicative of a role for natural selection in geographic expression differentiation.

**Table 3:** Lineage-specific expression divergence in *D. simulans*

Comparison	Population	LED	*t*-Test
All testis	Maine	0.2944	1.44E−01
All testis	Panama	0.2839	
All AG	Maine	0.3854	6.76E−45
All AG	Panama	0.5145	
Testis DE	Maine	0.4219	6.75E−42
Testis, not DE	Maine	0.1447	
Testis DE	Panama	0.4765	4.21E−52
Testis, not DE	Panama	0.1386	
AG DE	Maine	0.4122	5.80E−15
AG, not DE	Maine	0.2796	
AG DE	Panama	0.8199	7.31E−63
AG, not DE	Panama	0.4187	
Testis biased	Maine	0.1263	1.49E−91
Not testis biased	Maine	0.3269	
Testis biased	Panama	0.1375	2.75E−57
Not testis biased	Panama	0.3122	
AG biased	Maine	0.3106	7.59E−02
Not AG biased	Maine	0.3870	
AG biased	Panama	0.3567	6.27E−04
Not AG biased	Panama	0.5178	

For both tissues, in both populations, we see significantly longer branch lengths amongst DE genes than non-DE genes, as expected. However, tissue-biased genes are generally associated with shorter branches in both tissues, consistent with either stronger stabilizing selection or less directional selection on the former (Table 3). Similar to *D. melanogaster*, DE genes in the *D. simulans* AG exhibit much greater branch lengths to Panama than to Maine (0.82 vs. 0.41, *t*-test *P*-value = 3.3e^−50^), and much greater heterogeneity than non-DE genes. Thus, the two species show similar patterns of latitudinal DE in terms of faster evolution in the Panama population than the Maine population, as well as similar patterns of directionality. The testis shows a modest increase in branch length toward Panama amongst DE genes (0.47 vs. 0.42, *t*-test *P*-value 0.05). Overall, the branch length data, when considered alongside the parallelism data, support the view that there are two major categories of AG-biased genes, one which tends to exhibit lower expression differentiation with latitude, perhaps due to stabilizing selection, and another that appears to experience rapid expression differentiation associated with local adaptation.

### Seminal Fluid Protein Gene Geographic Expression Differentiation

Our previous analysis of Maine and Panama AG transcriptomes in *D. melanogaster* revealed a strong enrichment for *Sfps* among the DE genes (Cridland et al. 2023). Experimental evidence bearing on *Sfp* status (Wigby et al. 2020) is much richer for *D. melanogaster* than for the other two species, making rigorous comparative analysis challenging. Here, we use two approaches to identify *Sfp*s in non-*D. melanogaster* species. Our first approach uses orthologs of *D. melanogaster Sfps* in *D. simulans* and *D. hydei* and assumes that genes orthologous to *D. melanogaster Sfps* also function as *Sfps* in the other two species (Tables 4 and S14). This assumption is sensible for *D. simulans* but is less likely to be true for all orthologs in the distantly related *D. hydei*. To the extent that assumptions about conserved *Sfp* function are violated, conclusions about shared patterns of *Sfp* DE among the three species would likely be conservative.

**Table 4:** Differential expression amongst *Sfps*

Species	Tissue	Expressed Sfps	Sfp, DE	Not Sfp, DE	Sfp, not DE	Not Sfp, not DE	Total	Sfps/DE (%)	Sfps/notDE (%)	Fisher's exact test
*D. melanogaster*	AG	253	73	725	180	7,045	8,023	9.1	2.5	6.55E−18
*D. simulans*	AG	228	25	903	203	7,168	8,299	2.7	2.8	1.00E+00
*D. hydei*	AG	231	76	1,492	155	5,964	7,687	4.8	2.5	6.20E−06
*D. melanogaster*	Testis	187	4	35	183	10,666	10,888	10.3	1.7	4.33E−03
*D. simulans*	Testis	106	10	747	96	9,630	10,483	1.3	1.0	3.45E−01
*D. hydei*	Testis	217	16	456	201	9,760	10,433	3.4	2.0	4.73E−02

We were able to identify 266 orthologs of *Sfps* in *D. simulans* and only 130 orthologs in *D. hydei*. The smaller number of *D. hydei* orthologs of the Wigby *Sfps* is likely attributable, at least in part, to the short length and rapid sequence evolution of these genes (Swanson et al. 2001), which makes orthology assignments more challenging (Ranz et al. 2024). Real underlying *Sfp* presence/absence variation (Mueller et al. 2004; Begun and Lindfors 2005; Wagstaff and Begun 2005; Hurtado et al. 2022) and expression variation (Cridland et al. 2020; Thompson et al. 2024) may also contribute to this pattern. Because the *D. hydei* orthologs represent a small and likely biased sample of Wigby *Sfps*, we sought a more complete portrait of *Sfp* latitudinal DE in this species by taking advantage of a set of *Sfps* experimentally identified in the closely related repleta group species, *Drosophila arizonae* (Matzkin et al. 2024). We found a total of 213 matches between *D. arizonae* Sfps and *D. hydei* genes (Table S15). This, combined with the Wigby *Sfps*, brings the total of *D. hydei Sfps* identified to 279 (Table S16).

As expected, more *Sfps* are expressed in the AG than in the testis for all three species (Table 4). Among the *Sfp*s, 73, 25, and 76 exhibited latitudinal DE in the AG of *D*. *melanogaster*, *D. simulans*, and *D. hydei*, respectively (Tables S14 and S15). As reported previously (Cridland et al. 2023), *Sfps* are greatly enriched among DE genes in the *D. melanogaster* AG (3.6-fold, Fisher's exact test, *P* = 6.55e^−18^) as well as the *D. hydei* AG (∼2-fold, Fisher's exact test, *P* = 6.2e^−6^). We see no enrichment in the *D. simulans* AG. About two-thirds of *D. melanogaster* AG-expressed Sfps have lower expression in Panama, whereas in *D. simulans* only 34% and in *D. hydei* only 44% have lower expression in Panama (Table 5). While nearly all (98%, Cridland et al. 2023) DE Sfps in *D. melanogaster* AG show lower expression in Panama, both *D. simulans* and *D. hydei* exhibit less DE Sfp directionality; 13 of 25 have higher expression in Panama for *D. simulans* and 21 of 34 have higher expression in Panama for *D. hydei*.

**Table 5:** LogFCs amongst *Sfps*

Species	Tissue	Sfp expressed	Sfp,+logFC	Not Sfp,+logFC	Sfp,−logFC	Not Sfp, −logFC	Total	% Sfp with −logFC	Fisher's exact test
*D. melanogaster*	AG	253	85	4,034	168	3,736	8,023	66.4	9.31E−09
*D. simulans*	AG	228	150	4,480	78	3,591	8,299	34.2	2.28E−03
*D. hydei*	AG	231	130	3,665	101	3,791	7,687	43.7	3.81E−02
*D. melanogaster*	Testis	187	146	5,522	41	5,179	10,888	21.9	1.45E−13
*D. simulans*	Testis	106	50	4,960	56	5,417	10,483	52.8	9.22E−01
*D. hydei*	Testis	217	91	5,086	126	5,130	10,433	58.1	2.35E−02

Among the 25 *D. simulans* orthologs of Wigby *Sfps* that were DE in the AG, at least one, *lectin-46Cb*, is associated with a reproductive functional inference from mutant or RNAi analysis; *lectin-46Cb* protein binds to sperm in the seminal receptacle (Singh et al. 2018). Among the 34 *D. hydei* orthologs that are DE in AG and homologous to Wigby *Sfps* are a few with functional information (based on mutant or RNAi phenotypes in *D. melanogaster*) on their role in reproduction. For example, the *antares* protein binds to sperm and functions in the Sex Peptide pathway (Singh et al. 2018). This is intriguing given that the *Sex Peptid*e gene appears to have been lost in the common ancestor of the *repleta* group of Drosophila (McGeary and Findlay 2020; Hopkins and Perry 2022), suggesting that *antares* functions differently in *D. hydei* or some (unknown) protein has subsumed the role of *Sex Peptide* in this species. *CG17575* influences long-term female receptivity, oviposition, and release of sperm from the seminal receptacle (Ram and Wolfner 2007; Singh et al. 2018). *Tep4* (Dostálová et al. 2017) functions in immunity. *Pde1c* is involved in male fertility (Morton et al. 2010).

We observed 187 (64%), 106 (40%), and 217 (78%) Sfps expressed in the testis of *D. melanogaster*, *D. simulans*, and *D. hydei*, respectively. Thus, the proportion of Sfps/Sfp orthologs that were testis-expressed in the three species varied considerably, with *D. simulans* apparently expressing a considerably smaller proportion in the testis than the other two species. *Sfps* were similarly enriched among the DE genes in the *D. melanogaster* testis (Fisher's exact test, *P* = 0.004), though not as significantly as in the AG, likely due to smaller sample sizes. There was no enrichment of DE *Sfps* in the *D. simulans* and marginal enrichment in *D. hydei* testis (Fisher's exact test, *P* = 0.047; Table 4).

Testis-expressed Sfp directionality exhibits the opposite pattern from the AG, with a much smaller percentage of *Sfps* showing lower expression in Panama in *D. melanogaste*r and over 50% with lower expression in Panama in the other two species (Table 5). Given the general pattern of consistency in directionality for both all genes and DE genes in these tissues, this difference is surprising. Overall, *D. melanogaster* stands out among the three species as exhibiting the strongest evidence for enriched *Sfp* DE, which is likely shaped by spatially varying selection (Cridland et al. 2023).

### Gene Ontology Analysis

We used gene ontology (GO) enrichment analysis to investigate possible biological correlates of expression differentiation and similarities or differences in enrichments across species. For *D. simulan*s and *D. hydei*, we used GO terms from *D. melanogaster*, restricting gene lists composed of *D. melanogaster* orthologs. We used GOrilla (Eden et al. 2009) to examine enrichment of GO terms in DE genes relative to all expressed genes in *D. melanogaster*. We found a general pattern of a greater number of enriched terms for AG-expressed DE genes than for testis-expressed DE genes for all three species. This testis pattern was true not only for *D. melanogaster*, where few genes were DE, but also for *D. simulans* and *D. hydei*, which exhibited many testis DE genes (File S1). While no enriched terms in the AG of *D. melanogaster* were shared with enriched terms from *D. simulans* and *D. hydei*, we did find that some enriched terms were shared in *D. simulans* and *D. hydei*. These terms were primarily associated with the plasma membrane (plasma membrane part, intrinsic component of plasma membrane) or organelles (intracellular non-membrane-bounded organelle and non-membrane-bounded organelle).

## Discussion

This work has expanded our understanding of parallel local adaptation by adding a substantially diverged species, *D. hydei*, to comparisons of two closely related species, *D. melanogaster* and *D. simulans*. In addition to being highly diverged from *D. melanogaster/D*. *simulans*, its reproductive biology is dramatically different, and unlike *D. melanogaster* and *D. simulans*, its ancestral ecology is cactophilic (Oliveira et al. 2012). However, as all three species are recent arrivals to North America (Sturtevant 1921; Lachaise et al. 1988; Lachaise and Silvain 2004), the system presents an opportunity to begin identifying potential factors that influence species-specific vs. more general patterns of local adaptation. The lack of significant shared ancestral variation and independent demographic histories of colonization imply that any shared patterns of local adaptation are genetically independent, though parallel genetic changes may still contribute to parallel phenotypes.

A strong generalization reported here is that the AG exhibits more latitudinal DE than the testis. This is particularly extreme in *D. melanogaster*, where the proportion of DE genes is 20-fold greater in the AG than in the testis. *Drosophila hydei* exhibits a roughly 4-fold greater proportion of DE genes in the AG, while *D. simulans* exhibits only a 1.5-fold greater proportion of DE genes in the AG compared with the testis. While the explanation for weaker latitudinal expression differentiation in testis than AG is unknown, one possibility is that much regulation of spermatogenesis occurs post-transcriptionally (White-Cooper et al. 1998), potentially making it more likely that spatially varying selection acts at this level rather than at the level of transcript abundance (Fan et al. 2025). Existing data comparing transcriptomes and proteomes of these two organs in flies suggest that the correlation is stronger for AG than for testis (Garlovsky and Ahmed-Braimah 2023), consistent with this speculation. Obviously, this hypothesis cannot explain the dramatic heterogeneity between species in testis expression differentiation.

Another generalization reported here is that *D. melanogaster* shows less phenotypic differentiation for both tissues than either *D. simulans* or *D. hydei*. The simplest model, wherein both genomic and phenotypic differentiation result from drift (and assuming expression variation is highly polygenic), predicts that species exhibiting more phenotypic differentiation should also exhibit more genomic differentiation. This simple model is inconsistent with the data—for example, *D. simulans* shows less genomic differentiation than *D. melanogaster* (Machado et al. 2016; Sedghifar et al. 2016), yet more expression differentiation. *Drosophila hydei* shows similar levels of genomic differentiation as *D. melanogaster* (Zhao and Begun 2017), yet shows dramatically more phenotypic differentiation. These discordant observations imply the action of selection, though additional population genomic and genetic analysis will be required to understand the relative roles of selection on genomic and phenotypic differentiation in this three-species system.

Three pieces of evidence strongly support the conclusion that spatially varying selection shapes AG transcriptomes in all three species. First, *D. hydei* and *D. simulans* exhibit a large excess of shared DE genes, which also exhibit strongly parallel directionality; this can only be explained by parallel local adaptation. Second, all three pairwise species comparisons of Maine vs. Panama logFC show significant interspecific correlations in the AG, though the strength of these correlations varies. Though *D. melanogaster* and *D. simulans* share relatively few DE genes in the AG, among the three species pairs, this pair shows the strongest logFC correlation. Thus, the parallelism in *D. melanogaster* and *D. simulans* appears to be dominated by many subtle expression differences. Alternatively, *D. simulans–D. hydei* parallelism exhibits both shared outliers and more general logFC correlations. One interpretation of this pattern is that *D. simulans* has cumulatively experienced more spatially varying selection than *D. hydei* and *D. melanogaster*, as it shares widespread small expression changes with *D. melanogaster* and both general correlations and shared outliers with *D. hydei*. *Drosophila melanogaster* and *D. hydei* show the least evidence for shared parallel adaptation. Another line of evidence supporting parallel local adaptation is that both *D*. *melanogaster* and *D. simulans* show greater expression divergence in the lineage leading to the Panama population than to the Maine population, and, as noted above, both exhibit a trend toward lower expression in Panama. Thus, the AG exhibits multiple modes of parallel adaptation in these three species. The strong parallelism between *D. simulans* and *D. hydei* is striking, given their divergence time and their heterogeneous ecologies, morphologies, and mating systems.

Several possible themes emerge from examination of the list of shared DE AG genes in *D. simulans* and *D. hydei*. First, a large number of ribosomal proteins, 26, are shared, consistent with the idea that high and low latitude populations in both species are making differential investments in translation. Also consistent with this idea, three elongation initiation factors are DE, as are multiple genes associated with the Golgi apparatus or secretion. At least five genes associated with circadian biology are DE in both species; *tim*, *nocte*, *cueball*, *Oamb*, and *Hsp83*. Ten genes associated with the term “transcription factor” exhibited DE in the AG of both *D. simulans* and *D. hydei*: *cbd*, *crol*, *tou*, *Jra*, *tim*, *Hipk*, *Glut4EF*, *MEP-1*, *Mad*, and *lilli*. Eight of these were DE in the same direction, with lower expression in Panama in both species. Interestingly, *Mad* regulates the BMP signaling pathway, which functions in *D. melanogaster* secondary cell biology (Leiblich et al. 2012; Corrigan et al. 2014; Redhai et al. 2016). *dpp*, which also functions in the BMP signaling pathway, is also DE, consistent with the idea of spatially varying selection on secondary cell biology in these species. Other genes functioning in transcriptional regulation that show DE are *prd* (known to regulate AG transcription; (Xue and Noll 2002), *scribbler*, *ko*, and *retn*.

While *D. simulans–D. melanogaster* parallelism is manifested more in Maine vs. Panama logFC correlations than in shared DE genes, the set of shared DE genes suggests some possible targets of selection. We find five shared DE transcription factors (Table S9), including *tim*, which was previously identified in *D. melanogaster* (Cridland et al. 2023) at a more stringent *P* < 0.05 cutoff. All five, *tim*, *Abd-B*, *Taf3*, *CrebA*, and *Pdp1*, were more lowly expressed in Panama for both species. *timeless* and *Pdp1* are circadian rhythm genes, further supporting the idea that some latitudinal expression variation is connected to AG circadian biology. Notably, *tim* is DE in all three species, and all three species express the light-sensing protein-coding gene, *cry* (Emery et al. 2000), in our AG transcriptomes. Overall, the data from these three species suggest the existence of a taxonomically widespread light and/or temperature-dependent peripheral clock in the Drosophila AG. Interestingly, *Abd-B* and *CrebA* regulate key functions of the AG; *Abd-B* regulates secondary cells (Gligorov et al. 2013), suggesting that secondary cell biology is influenced by spatially varying selection in all three species, but with only partially overlapping genes. *CrebA* regulates the canonical secretion machinery (Johnson et al. 2020).

While the set of 14 three-species shared DE genes (Table S6) in the AG is not more than expected by chance, their associated biology may still be useful in hypothesis generation regarding processes under selection in all species. Most of these shared genes, 10 of 14, show the same directionality in all 3 species, a small, but significant bias. Among them are transcription factors *timeless* (Myers et al. 1996), and *knockout*. The gene *shep* exhibits clinal variation in North America (Roy and Castillo 2024), and though it has no known function in the AG, it was implicated by GWAS analysis as influencing circadian biology (Harbison et al. 2019). Females mutant for *Nep2* exhibit sperm storage phenotypes (Sitnik et al. 2014); this gene shows inconsistent expression directionality; greater expression in Panama in *D. simulans* and lower expression in Panama in *D. melanogaster* and *D. hydei* (Table S6). There is no evidence of a male mutant reproductive phenotype.

While there is much evidence in support of adaptive parallelism, there are also interesting differences between species. For example, the closely related species, *D. simulans* and *D*. *melanogaster,* differ by roughly 20-fold in testis latitudinal expression differentiation, strongly suggesting that the colonization of North America has been associated with very different evolutionary consequences for this organ. Seeking possible explanations for why such two closely related species are evolving in such different manner, we focus on one of the most obvious species differences potentially related to spermatogenesis—*D. simulans* segregates three different sex-ratio X syndromes, while there is no evidence for strong sex-ratio X chromosomes in *D. melanogaster* (but see [Reed et al. 2005; Corbett-Detig and Hartl 2012] for evidence of a weak distorter). These sex-ratio X systems are associated with evolution of novel genes emerging by duplication and influencing spermatogenesis (Tao et al. 2007; Muirhead and Presgraves 2021; Vedanayagam et al. 2021). At least one of the sex-ratio X chromosomes has a temperature-sensitive phenotype (Tao et al. 2007), consistent with the possibility of spatially varying selection. Unfortunately, there are no estimates of the frequency of *D. simulans* sex-ratio X chromosomes in any North American populations. There are no reports of sex-ratio X chromosomes in the repleta group of Drosophila, so it seems unlikely that high levels of testis expression differentiation in *D. simulans* and *D*. *hydei* would both be explained by sex-ratio conflicts. Another speculative hypothesis for the difference in rates of testis expression differentiation between *D. melanogaster* and *D. simulans* is differences between species in recent transposable element activity associated with their different colonization histories (Vieira et al. 1999; Biémont et al. 2003; Kofler et al. 2015; Scarpa et al. 2025). While it is natural to seek explanations for major differences between closely related species, an equally valid question is, “Why does *D. melanogaster* exhibit such low levels of testis expression differentiation compared to the other two species”?

Another clear difference between species is the contribution of AG-expressed testis-biased genes and seminal fluid protein genes to latitudinal expression differentiation in the AG. For example, *D. melanogaster* and *D. hydei* show strong enrichments of Sfps among DE genes, while *D*. *simulans* shows very little. *Drosophila melanogaster* and *D. simulans* show very strong logFC correlations for testis-biased genes in the AG, while other species pairs show no obvious difference between testis-biased and non-testis-biased orthologs. Finally, the three species exhibit different degrees of expression of testis-biased genes in the AG, suggesting that the set of testis-biased genes that are also expressed in the AG may evolve fairly quickly. Establishing the functional significance of testis-expressed *Sfps* will be necessary to generate hypotheses on these population and evolutionary phenomena.

Clarifying the possible factors explaining the patterns of parallelism documented here will be challenging. Integration of population genomics data with genetic, epigenetic, and organismal phenotypic analysis of AG function in all three species may provide clues as to why adaptive parallelism is distributed across species in a heterogeneous and idiosyncratic fashion. Regardless of the agents of selection, identifying the trans- and cis-acting variants generating expression parallelism will provide insights as to whether phenotypic parallelism reported here is reflected by underlying genetic parallelism.

## Materials and Methods

### Flies

The population samples for all three species were previously described (Zhao et al. 2015; Zhao and Begun 2017) and were collected from Fairfield, Maine (September 2011, Latitude: 44°37′N) and Panama City, Panama (January 2012, Latitude: 8°58′N). After establishment, isofemale lines were maintained at 25 °C on a standard yeast-cornmeal-agar food. Testis and AG tissue were isolated from sexually mature, virgin males. As in our previous work, we included the anterior ejaculatory duct in these dissections, but for simplicity, we will refer to the dissected somatic tissue as AG. Males were collected within 3 h of eclosion and aged in groups prior to dissection. *Drosophila melanogaster* and *D. simulans* males were aged 3 to 5 d. *Drosophila hydei* males were aged to 14 to 16 d before dissection, as they do not become sexually mature for at least 10 d after eclosion (Markow 1985). We used 12 Panama strains and 12 Maine strains for *D. melanogaster*, 13 Panama strains and 13 Maine strains for *D. simulans*, and 13 Panama strains and 12 Maine strains for *D. hydei.* Pools of dissected tissues were created by sampling the tissue for three males from each isofemale line from a population to create a pool of dissected tissue—with equal contribution from each line—representing a replicate of a population. We generated three independent replicates for each of two populations and tissues, and all three species. One testis pool from *D. melanogaster* was found to be an outlier (Fan et al. 2025) and was dropped for a total of 35 libraries used in these analyses (Table S1). We used *D. simulan*s individuals from four lines from Madagascar and three lines from Zimbabwe, Africa, to root the network used to estimate branch lengths to Maine and Panama. The *D. melanogaster* transcriptomic data reported here were previously described in Cridland et al. (2023). The *D. simulans* transcriptomic data were described in Fan et al. (2025).

### RNA Extraction and Sequencing

Tissues were dissected into cold phosphate buffered saline, transferred into cold Trizol, and stored at −80 °C until RNA extraction. Tissues were homogenized in 200 μl Trizol and the Trizol volume adjusted to 1 ml; 200 μl of chloroform was added and the tube was shaken for 20 s, followed by incubation for 5 min at room temperature. Samples were then centrifuged at 4 °C and 13,000 rpm for 15 min, and the upper phase was collected. After the addition of 1 μl glycogen, 500 μl isopropanol was added, followed by mixing by gentle inversion. Samples were left at −20 °C for 1 h, after which nucleic acids were pelleted and then washed with 70% ethanol, followed by drying and resuspension in nuclease-free water. All samples were subjected to DNase digestion using the TURBO DNA-free kit (Ambion) following the manufacturer's protocol. DNase digestion was performed with the TURBO DNA-free kit (Ambion) using the manufacturer's protocol; samples were cleaned up with HighPrep RNA Elite beads (MagBio Genomics). The qualities of the resulting RNAs were assessed on a Bioanalyzer (Agilent). After fragmentation, first-strand synthesis was carried out via random hexamer priming, followed by second-strand synthesis. After end repair and A-tailing, adaptors were ligated, followed by size selection, amplification, and purification. Library qualities were estimated using the High Sensitivity DNA chip on a Bioanalyzer (Agilent); 150-bp paired-end reads were generated on an Illumina NovaSeq 6000 machine.

### Assemblies and Annotations

The following genomic resources were used for analysis of RNA-seq reads: *D. melanogaster* genome version 6.41 (www.FlyBase.org), *D. simulans* genome *v*ersion 3.1 (GCF_016746395.2), and *D. hydei* GCF_003285905.1. We downloaded annotation information for each gene from NCBI (*D. hydei* downloaded 2021 September 16, *D. simulans* downloaded 2023 September 5) and FlyBase (*D. melanogaster* downloaded 2021 August 9). For characterizing patterns of variation in each species, we use all expressed orthologs regardless of the identification of orthologs in the other two species. However, most analyses use pairwise 1:1 orthologs or the three-species 1:1:1 orthologs. We used the gene annotation information downloaded from NCBI to identify previously annotated orthologs between *D. simulans* and *D. melanogaster.* We also used previous manual annotation of some *D. simulans* seminal fluid proteins (Majane et al. 2024). To expand the set of orthologs between *D. melanogaster* and *D. simulans,* we then identified reciprocal best hits between proteins using blastp (-evalue 1e−10). We then used tblastx (evalue 1e−10) to identify orthologs for genes not identified with blastp. To find orthologs between *D. melanogaster* and *D. hydei* we first identified reciprocal best hits between proteins followed by reciprocal best hits between transcripts as was done for the *D. melanogaster* vs. *D. simulans* comparison. Following the pairwise comparisons between the three species, we performed a three-way analysis of all protein sequences using mcl (van Dongen and Abreu-Goodger 2012), which uses a Markov cluster algorithm for assigning genes into families. The inflation value was set to 4 after evaluating the clusters produced at a variety of cluster granularity settings. Sets of 1:1:1 orthologs were identified and used to further identify orthologs not identified by the reciprocal best hits approach.

### Gene Expression Analysis

Reads from each species were aligned to the corresponding reference genome assemblies and annotations using Hisat2 (Kim et al. 2015) with default parameters. StringTie (Pertea et al. 2015) was used to calculate TPM for each gene in each population transcriptome for each species. For each species we retained “expressed” genes, defined as those exhibiting median TPM > 1 in at least one of the two populations. Similarly, for comparisons of DE genes between species, we required that a gene was categorized as “expressed” in both (or all species in the case of the three-way species comparison). Read counts per gene were generated with featureCounts using the paired-end read option (Liao et al. 2014). Differential expression between Maine and Panama was measured for each species with limma (Ritchie et al. 2015). As our primary goal here is investigating species parallelism, most analyses focus on an adjusted *P*-value cutoff of ≤0.1 rather than the more conservative adjusted *P*-value cutoff of 0.05, the logic being that a slightly more liberal false discovery rate is very unlikely to generate spurious parallel population differentiation.

### Tissue-Biased Gene Identification

To investigate possible connections between tissue-biased expression and geographic expression differentiation, we first used the FlyAtlas2 (Leader et al. 2018) resource to identify either AG or testis-biased genes in *D. melanogaster.* We calculated tau (*τ*) (Yanai et al. 2005) from the set of male tissues in FlyAtlas2 and considered any gene with a *τ* of ≥0.9 to be biased in the tissue where it was expressed most highly. Since there is no comparable resource for either *D. simulans* or *D. hydei* we could not calculate *τ* similarly for these species. Instead, to enable appropriate comparative analyses we compared whole male data to AG and testis data separately for each species (including *D. melanogaster*) using limma (Zhao et al. 2015; Zhao and Begun 2017), using the *D. melanogaster* tissue data from FlyAtlas2 to investigate the parameters of organ vs. whole male *D. melanogaster* data were strongly correlated with the estimates of *τ* from the same dataset using all adult male tissues. In other words, we used FlyAtlas2 data to “calibrate” an organ vs. whole male analysis that approximated the tissue-based estimate and then used this for all three species. We investigated the effects of variation in log fold change, adjusted *P*-values, average expression (from limma), and TPM (calculated from StringTie) for genes identified as either AG or testis-biased based on our analysis of the FlyAtlas2 data.

For AG we chose a log fold change of −3.59 in the AG vs. whole male, corresponding to the third quartile of the distribution for genes identified as biased based on the FlyAtlas2 tissue comparison. We also picked an adjusted *P*-value of ≤0.01, a minimum TPM of ≥1, an average expression, a value calculated post-normalization in limma, of >0, and a log fold change ≥1 in the testis vs. whole male. For the testis comparisons we used a log fold change of −0.46 in the testis vs. whole male, an adjusted *P*-value of ≤0.01, a minimum TPM of ≥5, an average expression ≥0, and a log fold change in the AG vs. whole male of ≥1. The values for both tissues were chosen to maximize the overlap between the tissue-biased genes identified via FlyAtlas2 and those identified using the cutoff criteria.

Applying these cutoffs to *D. melanogaster* organ vs. whole male data returned a gene list largely overlapping the list identified as tissue-biased using all the FlyAtlas2 male tissue data (84% AG, 80% testis), though there were some additional genes in these organ vs. whole male lists not present in the all-tissue-based *τ* estimate. We then applied the same parameters to our *D. simulans* and *D*. *hydei* organ vs. whole male data. Note that because of the close phylogenetic relationship between *D. melanogaster* and *D. simulans* and the similar sizes of the body and organs, we expect this approach to generate a highly correlated set of AG- and testis-biased genes in the two species, with differences between species most likely resulting from gene expression divergence in the body or organs. Alternatively, we are less certain this is the case for *D. hydei*, which compared with *D. melanogaster* and *D. simulans,* has a bigger body, bigger AG and testis, and a somewhat different composition of cell types in the AG (Takashima et al. 2023). Nevertheless, using the same approach on all three species may still provide biologically useful information about AG- or testis-biased expression and its connection to latitudinal expression differentiation.

### Branch Length Analysis

We calculated a four-population branch length statistic (Cridland et al. 2023) using median TPMs for each population following (Jiang and Assis 2020) for the *D. simulans* Maine and Panamanian populations. Branch lengths to Maine and Panama (AG and testis were analyzed separately) were estimated using individuals from Madagascar and Zimbabwe to root the network (Table S1).

### GO Analysis

We used Gorilla (Eden et al. 2007, 2009; http://cbl-gorilla.cs.technion.ac.il/) to identify GO terms that were enriched among DE genes, using a background of all genes expressed in the tissues of interest (mean TPM > 1 in Panama, Maine, or both) and the default *P*-value threshold. We performed this analysis for DE genes identified in *D. melanogaster* as well as for the *D. melanogaster* orthologs of genes found to be DE in *D. simulans* and *D. hydei*.

### Comparison to *D. arizonae* Seminal Fluid Protein Genes

We obtained genomic sequences and annotations for *D. arizonae* from (https://cactusflybase.arizona.edu/, downloaded 2025 March 14). We used a reciprocal best hit BLAST analysis between *D. arizonae* and *D. hydei* transcripts to identify a list of *D. hydei* orthologs of *D. arizonae* seminal fluid protein (*Sfp*) genes reported in Table S6 of (Matzkin et al. 2024). Given that both D. *hydei* and *D. arizona* are *repleta* group species, we make the simplifying assumption that genes experimentally identified as *Sfps* in *D. arizonae* also function as *Sfps* in *D. hydei*.

## Supplementary Material

evaf161_Supplementary_Data

## Data Availability

Sequencing data are available on https://ncbi.nlm.nih.gov/sra under PRJNA1171453, PRJNA890704, and PRJNA1273198.
