# Supplementary material for: Phylogenetic Relatedness Does not Predict Patterns of Parallel Transcriptome Adaptation in Drosophila
Source: Genome Biol Evol. 2025 Aug 19;17(9):evaf161. doi: 10.1093/gbe/evaf161 (PMC12448186; doi:10.1093/gbe/evaf161)
Supplement: evaf161_Supplementary_Data [file evaf161_supplementary_data.zip › Figure Legends.docx]

Figure Legends:

Figure S1: Pairwise comparisons of logFC between species in the testis. For panels A-C shared differentially expressed genes are in red, all other genes are in black. A) *D. melanogaster* vs. *D. simulan*s B) *D. melanogaster* vs. *D. hydei* C) *D. simulan*s vs *D. hydei*.

Table S1:Libraries

Table S2: Number of Genes Expressed at TPM ≥ 1

Table S3: Differentially Expressed Genes

Table S4: Shared DE Between Species

Table S5: List of Shared DE Genes

Table S6: Three-way Shared DE Genes Expressed in the AG

Table S7: Shared Directionality for DE Genes

Table S8: logFC Correlations Between Species

Table S9: Transcription Factors with Shared DE

Table S10: Orthologs of Tissue Biased Genes in *D. melanogaster* Identified Using FlyAtlas2 Data

Table S11: Tissue Biased Genes per Species

Table S12: Biased Genes Expressed in the AG

Table S13: Shared Testis Biased Genes in the AG

Table S14: *D. melanogaster* *Sfps* per Species

Table S15: *D. arizonae* Identified *Sfps* in *D. hydei*

Table S16: *D. melanogaster* / *D. arizonae* Overlapping *Sfps*
